# The skin microbiome in psoriatic disease: A systematic review and critical appraisal

**DOI:** 10.1016/j.jtauto.2019.100009

**Published:** 2019-08-20

**Authors:** Meital Yerushalmi, Ofir Elalouf, Melanie Anderson, Vinod Chandran

**Affiliations:** a Psoriatic Disease Program, Krembil Research Institute, University Health Network, Canada; b Institute of Medical Science, University of Toronto, Canada; c Laboratory Medicine and Pathology, University of Toronto, Canada; d Division of Rheumatology, Department of Medicine, University of Toronto, Canada; e Library and Information Services, University Health Network, Canada

**Keywords:** Skin microbiome, Psoriasis, Psoriatic arthritis, Systematic review, Next-generation sequencing

## Abstract

**Background:**

Psoriasis affects 1–3% of the Canadian population. Psoriatic arthritis (PsA), the most common comorbidity of psoriasis, affects up to 30% of psoriasis patients. The skin microbiome is hypothesized to play a role in the pathogenesis of psoriatic disease (PsD-psoriasis and PsA).

**Objective:**

To summarize the current state of literature on the skin microbiome in PsD.

**Methods:**

A systematic review was performed using searches in Ovid, Medline, Embase, Medline Epub Ahead of Print and In-Process & Other Non-Indexed Citations, and Cochrane Central Register of Controlled Trials (CENTRAL). Search was limited to humans and English language, with no limits for date or publication type.

**Results:**

Of 4,032 citations identified, 9 studies met inclusion criteria (7 on psoriasis only and 2 studies compared the microbiome characteristics between psoriasis and PsA). Compared to healthy controls, lesions demonstrated a decreased alpha diversity, higher relative abundances of Firmicutes, and lower relative abundances of Actinobacteria. Less conclusive were genus-level results, which nonetheless demonstrated trends towards increased *Streptococcus*, *Staphylococcus*, and *Corynebacterium* and decreased *Propionibacterium* in lesions vs. control.

**Limitations:**

Study designs were heterogeneous, including sampling technique and exclusion criteria.

**Conclusions:**

Phyla- and selected genus-level characteristic of the psoriatic microbiome are presented; further research is warranted.

## Highlights

• Psoriatic lesion demonstrates decreased alpha diversity compared to healthy skin.
• Relative abundance of Firmicutes is higher in psoriatic compared to healthy skin.
• Relative abundance of Actinobacteria is lower in psoriatic compared to healthy skin.
• Studies vary in methodology, including sampling technique and exclusion criteria.

## 1 Introduction

### 1.1 Psoriasis and psoriatic arthritis

Psoriasis is a common immune-mediated inflammatory skin disease affecting 1–3% of the Canadian population [1,2]. It is characterized by sharply demarcated, erythematous, indurated plaques covered by silvery-white scales, as well as systemic comorbidities [3,4]. Psoriatic arthritis (PsA) is an inflammatory arthritis that affects up to 30% of psoriasis patients [5]. The manifestations and comorbidities of psoriasis and PsA, considered together as psoriatic disease (PsD), negatively impact a patient’s quality of life [3].

### 1.2 The skin microbiome

The skin is the body’s first line of defense against toxic substances and pathogens, with an arsenal of immune cells and antimicrobial mediators [6]. It lies in close proximity to the skin microbiome, the collection of microorganisms residing on it, placing the microbiome at an optimal interphase to educate the immune system to tolerate resident microorganisms while being able to respond effectively against pathogens. In addition, the skin microbiome confers numerous benefits to the host, including resisting pathogen colonization, maintaining the skin barrier, and modulating the inflammatory response [7]. Given its role in cutaneous immunity, it is not surprising that the skin microbiome has been investigated in psoriasis. Importantly, the cutaneous microbiome composition varies based on anatomical locations classified into microenvironments [section 4.3], any of which could be affected by psoriasis.

### 1.3 The skin microbiome in psoriasis and psoriatic arthritis

Though several groups have studied the cutaneous microbiome of a psoriatic plaque, the role of skin bacteria in psoriasis is still not well-understood. Differences in their results may stem from variation in study design and methodology, including differences in sampling and processing techniques. This systematic review summarizes the literature on the microbiome in PsD and critically examines study methodologies to identify biases and gaps in knowledge to be addressed in future studies.

## 2 Methods

### 2.1 Search strategy, exclusion/inclusion criteria, and data extraction

A systematic search of the literature in Ovid databases Medline, Embase, Medline Epub Ahead of Print and In-Process & Other Non-Indexed Citations, and Cochrane Central Register of Controlled Trials (CENTRAL) was performed on 20 April 2017 and on 24 January 2018 using subject headings and keywords including terms for PsD, for bacteria or microbiota, and for skin (Supplementary Appendix 1, eTables 1–3: https://dx.doi.org/10.17632/crhb9gdgbj.2) by an experienced librarian (MA). No date limits were set, and studies in languages other than English or those involving non-human subjects were excluded. Two authors (MY and OE) screened all resulting titles, and subsequently screened relevant abstracts and full-text articles for eligibility according to the inclusion and exclusion criteria (Supplementary eMethods: https://doi.org/10.17632/crhb9gdgbj.2). Studies utilizing culture-independent, targeted (16S rRNA) sequencing of the psoriatic bacterial microbiome in psoriasis or PsA were included. Studies investigating other dermatological diseases (which may be associated with a specific microbiome signature) were excluded, as were those employing culture-based methodology (due to the bias toward representing bacteria that thrive in lab culture conditions), metagenomic sequencing, or mass spectrometry (as most studies have been conducted with targeted sequencing). Two authors (MY and OE) extracted data using a standardized data extraction form and assessed the risk of bias using a Risk of Bias Analysis Tool (adapted from Hamidi et al. [8]) (Supplementary eTable 4: https://dx.doi.org/10.17632/crhb9gdgbj.2). Bias domains were selected to reflect relevant factors in general and skin microbiome research as well as clinical psoriasis evaluation. Disagreements on study eligibility and data extraction were resolved by a third author (VC).

## 3 Results

### 3.1 Search results

**Fig. 1:**
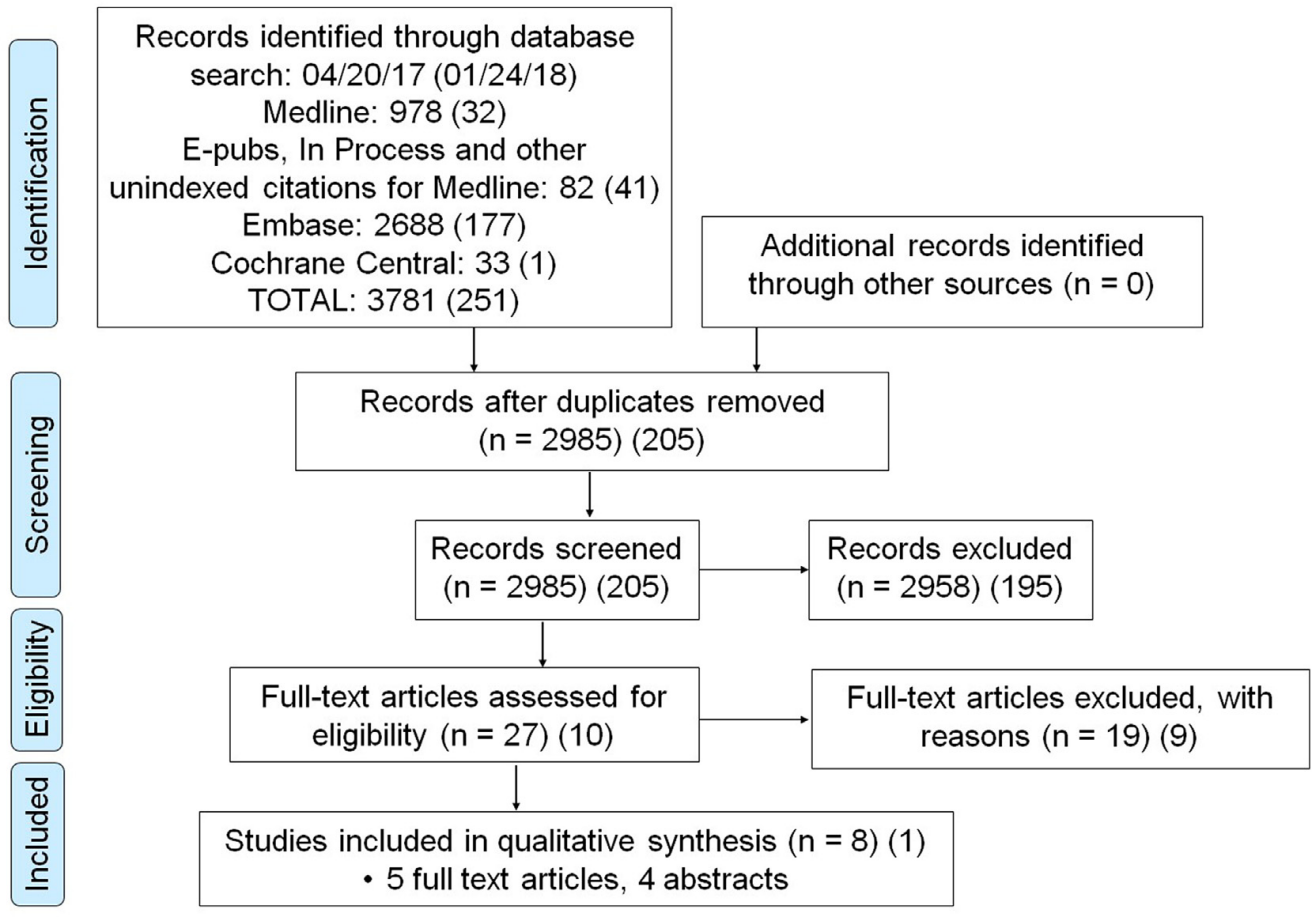
Systematic review flow diagram.

In total, 4,032 studies were identified (Fig. 1), of which 3,190 were screened after removing duplicates. Of those, 37 were assessed for eligibility in full-text, and 9 studies included in the review based on the selection criteria (5 full-text articles and 4 studies published as abstracts only). Due to heterogeneity in included studies’ population, design, and methods, qualitative rather than quantitative synthesis was carried out.

### 3.2 Article selection and characteristics

Nine studies were identified (Table 1): 8 were cross-sectional (of which 1 study included a longitudinal subset analysis following the use of anti-inflammatory drugs) and 1 was interventional. Of these 9 studies, 2 abstracts included both psoriasis and PsA patients while the remaining studies focused on psoriasis only. Altogether, 7 studies included 155 psoriasis patients and 74 PsA patients (88 males and 42 females, mean [standard deviation] age 50 [5.5] years; 2 studies did not report this data [[9], [10], [11], [12], [13], [14], [15]]). Chronic plaque psoriasis was specified and assessed for severity in 5 of the studies, albeit with different clinical outcome measures (4 with Psoriasis Area and Severity Index [PASI] [9,11,14,15] and 2 with Body Surface Area [BSA] [9,13]). Psoriasis was considered mild in 2 [[9], [14]] and moderate-to-severe in 3 [[11], [13], [15]] of the studies. Two studies implemented an intervention and examined microbiome composition based on psoriasis severity [9,15]. Varied sampling procedures were employed, including punch biopsy [12], curettage [11], cotton pledget [9,13,14], and commercial swab [10,[15], [16], [17]].

**Table 1:** Description of studies included in the systematic review.

	Study design		Number of psoriasis patients (PsA patients)	Patients’ average age	Patients’ sex ratio (M:F)	Psoriasis type	Psoriasis severity (mean ± SD)	Type of sample
	Cross sectional	Pre-post intervention						
Martin et al. [15]		X	27	59.9 ± 11	17:10	Chronic plaque psoriasis	Initial PASI: 21.2 ± 10.8	Swab
Alekseyenko et al. [9]	X	X	51	49.1 ± 16.4	39:12	Chronic plaque psoriasis	Mean PASI: 8.7 ±10.1; BSA: 9.4 ±13.9	Swab
Fahlén et al. [12]	X		10	Age range: 24–60 years	5:5	Chronic plaque psoriasis	Unspecified	Punch biopsy
Drago et al. [11]	X		1	50 ± 3 (all subjects)	1 M	Unspecified	PASI: 20	Curettage
Gao et al. [13]	X		6	46.3 ± 15.7	3:3	Unspecified	BSA: 12 ± 5.7	Swab
Langan et al. [14]	X		14	51 ± 12.2	9:5	Chronic plaque psoriasis	PASI 5.1 ± 3.9	Swab/washing
Yan et al. [16]	X		8	Unspecified	Unspecified	Unspecified	Unspecified	Swab
Castelino et al. [10]	X		9 (12)	48 (PsC) 56 (PsA)	5:4 (PsC) 9:3 (PsA)	Chronic plaque psoriasis	Unspecified	Swab
Manasson et al. [17]	X		29 (62)	Unspecified	Unspecified	Unspecified	Unspecified	Swab

### 3.3 Study design & methodology

#### 3.3.1 Study design: sample site selection & exclusion criteria

Several elements of study design and methodology were compared across studies (Supplementary Table 1: https://doi.org/10.17632/crhb9gdgbj.2). Lesion sample sites included the extensor aspect of the knee [9,13,15] and elbow [9,11,15], the back [9,[12], [13], [14], [15]], and the posterior auricular crease [9,11,14]. Five of the studies obtained lesional samples from more than one microenvironment [9,[12], [13], [14], [15]], of which two studies subsequently grouped samples according to body region but not, in all cases, according to the microenvironment [9,12]. Unaffected samples (healthy skin in a psoriasis patient) were included in 7 studies and obtained from a site closest to lesion [11,15], contralateral to lesion [9,17], or from other sites [13]. Atopic dermatitis patients were included in two of the studies [11,15]. Exclusion criteria varied as only 3 studies specifically excluded antibiotic use [9,11,14] and topical psoriasis treatment [[9], [10], [11], [12], [13], [14], [15]], and 2 excluded UV therapy [12,13].

#### 3.3.2 Study methodology: DNA extraction kits and sequenced region of the 16S rRNA gene

The most commonly used DNA extraction kits were Qiagen DNeasy® Powersoil® Kit (formerly MoBio PowerSoil® DNA Isolation Kit) [10,15,17] and Qiagen DNeasy® Blood and Tissue Kit [9,12,13]. Most studies utilized high-throughput sequencing platforms targeting different regions of the 16S rRNA gene (V1-V2 [15], V1-V3 [9], V3-V4 [[10], [12]], V4 [17], V2-4-8 and V3-6, V7-9 [11]).

### 3.4 Taxonomic analysis

Analysis of results was performed at various taxonomic levels including phyla [9,[11], [12], [13], [14], [15], [16]], class [9], order [9], family [9,11], genus [9,[11], [12], [13], [14], [15], [16]], and species/OTU [9,11,13,16]. Owing to their link to disease mechanism and severity, *Streptococcus* and *Staphylococcus* genera results were presented in several studies [9,12,13,15,17]. Nevertheless, considering the variation in taxonomy level at which results are presented, we resorted to reporting shifts in relative abundances in the four most common phyla in human skin, reported by all full-text articles.

### 3.5 Psoriatic plaque microbiome profile

#### 3.5.1 Alpha and beta diversity

Table 2 describes the diversity, unaffected site characteristics, and relative abundances of the four most common phyla in the psoriatic plaque. Of the 7 studies that focused on psoriasis microbiome (as opposed to PsA), 4 reported lower alpha diversity values in lesional skin compared to control [9,12,14,16] while 1 study reported similar values [15] and another reported higher diversity in the lesion [13]. No trends in beta diversity were found, with some studies demonstrating higher values in lesions than controls (not statistically significant [9]) and others demonstrating the opposite (significance unspecified [12]) or significant differences that were nonetheless unspecified [14]. PsA abstracts reported no significant difference in alpha diversity between PsA and psoriasis without PsA (PsC) [10,17]. In contrast, beta diversity results vary among studies and account for little [10] or no [17] variance between PsA and PsC. Similarly, unaffected skin characteristics varied, with 2 studies concluding that they were similar to lesional samples [15,16], 2 to skin samples from healthy controls [11,13], and 1 concluding intermediate microbiome profile between lesional and healthy control samples [9].

**Table 2:** Psoriatic plaque microbial diversity and taxonomic characteristics.

	Diversity	Unaffected sample	Relative phyla abundance			
			Proteobacteria	Firmicutes	Actinobacteria	Bacteroidetes
Martin et al. [15]	α-diversity: similar between L, U	Similar to L	C > L,U^a^	L,U > C^a^	L,U > AD^a^	No difference between L, U
Alekseyenko et al. [9]	α-diversity: C > U > L β-diversity: L > U > C (not significant)	Intermediate between L and C	C > L^a^,^b^	L > C^b^	L > C^b^	C > L^a^,^b^
Fahlén et al. [12]	α-diversity: C > L (not significant) β-diversity: C > L^a^	N/A (no U samples)	L > C (trunk)	No difference (limb or trunk)	Overall: C > L Limb or trunk: no difference	Not reported
Drago et al. [11]	N/A	Similar to C	L > C,AD^a^	C,AD > L^a^	No difference between L, C, AD^d^	L > C,AD^a^^,^^d^
Gao et al. [13]	α-diversity: L > U,C	Similar to C	C > L^c^	L > U,C	C,U > L	L > C,U^a^
Langan et al. [14]	α-diversity: C > L,U^a^ β-diversity: significant difference between L, U (unspecified)	Significantly differ from L		L > C	C > L	
Yan et al. [16]	α-diversity: C > L	Similar to L		L > C	C > L	

L = lesion, U = unaffected, C = healthy control; AD = atopic dermatitis.

Cells were left empty if information regarding the relative abundance of the respective phylum was not mentioned in the paper.

a Significance level unspecified.

b Dominate a cutaneotype enriched in lesion or control samples.

c Detection frequently between lesion and control samples.

d Derived from figure only.

#### 3.5.2 Relative abundance

##### 3.5.2.1 Phyla level

Relative abundances of microbial phyla were presented as percentages in four of the five full-text studies and were estimated from a figure in the remaining study [11]. To allow for amalgamation of results, relative abundances were inferred from reported phyla percentages, phyla domination of cutaneotypes enriched in either lesion or control samples, and phyla detection frequencies among the different groups. Overall, most studies reported that psoriatic lesions are characterized by higher relative abundances of Firmicutes [9,[13], [14], [15], [16]] and lower relative abundances of Actinobacteria [[12], [13], [14],^16^] compared to controls. Conflicting results between studied precluded concluding trends on Proteobacteria and Bacteroidetes.

##### 3.5.2.2 Lower taxonomic levels

Microbiome characteristics of the psoriatic plaque at lower taxonomic levels were not clear (Supplementary Table 2: https://doi.org/10.17632/crhb9gdgbj.2). The levels of taxonomy at which differences in relative abundance were reported varied from Class and Order [9] to Family [9,11], though no taxon was reported by more than a single study as different between psoriatic, unaffected, and healthy control samples. Genus-level results were reported by all full-text studies [9,[11], [12], [13],^15^] and two abstracts [16,17]. Most studies reported that the relative abundances of genera *Streptococcus* [12,13,16], *Staphylococcus* [13,15], and *Corynebacterium* [13,15] were increased in lesional vs. control samples. In contrast, Fahlén et al. [12] reported an opposite, significant effect with *Staphylococcus* (limb samples only). In addition, a trend of decreased *Propionibacterium* in lesion vs. control was reported [12,13,16], although Alekseyenko et al. showed no difference in its abundance between the groups. Consistent with this trend, *Streptococcus/Propionibacterium* ratios were reported to be significantly higher in lesions vs. control [12,13]. While additional genera were reported as differing in relative abundance between lesional and control samples [9,16], none were common between the studies. Lastly, several studies reported species- or OTU-level results [9,11,13,16], including a decrease in lesional *Propionibacterium acnes* compared to control [11,13].

### 3.6 Risk of bias

Table 3 reports the results of the risk of bias analysis. Only full-text articles were assessed for risk of bias, as not all criteria are presented within the limited scope of an abstract. The quality of studies varied as reflected by resultant risk-of-bias score and estimate: low [9], moderate [13,15], and high [11,12]. The main factors increasing bias were lack of site- and microenvironment-based matching between lesion and control samples, no exclusion of treatment relevant to skin microbiome, and absence of false-discovery rate application as part of the analytical assessment.

**Table 3:** Analysis of study domains that may create a bias in results.

Bias Domain	No risk of bias if:	Martin et al. [15]	Alekseyenko et al. [9]	Fahlén et al. [12]	Drago et al. [11]	Gao et al. [13]
Sample selection	Consecutive, unselected population of patients	N/A	N/A	N/A	N/A	N/A
	Control samples obtained from healthy subjects	Yes	Yes	No	Yes	Yes
Confounding factors	Age-matched psoriasis, control subjects (±5 years)	N/A	Yes	N/A	Yes	No
	Sex-matched psoriasis, control subjects	N/A	Yes	Yes	Yes	No
	Unaffected sample contralateral	No	Yes	N/A	No	No
	Site-matched psoriasis, control samples	Yes	No	No	Yes	No
	Microenvironment-matched psoriasis, control samples	No	No	No	Yes	No
	Antibiotics excluded	No	Yes	No	Yes	No
	Topical medications excluded	No	No	Yes	Yes	Yes
	UV therapy excluded	No	No	Yes	No	Yes
Exposure assessment	Psoriasis diagnosed by a dermatologist	Yes	Yes	No	Yes	No
	Psoriasis type specified	Yes	Yes	Yes	No	No
	Psoriasis severity assessed	Yes	Yes	No	Yes	Yes
Attrition bias	Reason(s) for subject exclusion reported	Yes	Yes	N/A	N/A	N/A
Selective outcome reporting	Alpha diversity reported	Yes	Yes	Yes	No	Yes
	Beta diversity reported	No	Yes	Yes	N/A	Yes
	Relative taxa abundances reported	Yes	Yes	Yes	Yes	Yes
	Significance of differences in taxa abundances reported	No	Yes	Yes	No	Yes
Analytical assessment	False discovery rate applied	No	Yes	No	No	No
	Use of a single test for the same outcome	Yes	Yes	Yes	Yes	No
Risk of bias^a^		Mod	Low	High	High	Mod

a Low - if No risk of bias in at least 5 of the 6 domains; Moderate - if No risk 3 or 4 domains; High - if No risk of bias in 2 or less domains.

## 4 Discussion

### 4.1 Summary of results

Our review demonstrates that psoriatic lesions are characterized by decreased alpha diversity [9,12,14,16], higher relative abundances of Firmicutes [9,[13], [14], [15], [16]] and lower relative abundances of Actinobacteria [[12], [13], [14],^16^] compared to healthy controls. While results at lower taxonomic levels are less conclusive, genera *Streptococcus* [12,13,16], *Staphylococcus* [13,15], and *Corynebacterium* [13,15] are increased in relative abundance while *Propionibacterium* is decreased in lesions vs. healthy control [12,13,16]. In addition, *Streptococcus/Propionibacterium* ratios are significantly higher and *Propionibacterium acnes* decreased in lesion compared to control. However, the heterogeneity between the studies and their varied risk of bias must be considered when interpreting the results. Next, we examine elements of study design that may affect the resultant characterization of the psoriatic plaque and should, therefore, be considered in future investigations.

### 4.2 Control and unaffected sample selection

The comparison of psoriatic lesions to control skin (healthy subject) or unaffected skin (healthy skin in a psoriatic patient) is valuable in establishing deviations from a healthy microbiome and exploring whether such changes are local or systemic. A valid control sample should, ideally, be free of potential biases that may affect its characteristics and be site-matched to the lesion sample. Nevertheless, owing to the nature of their lesion samples (biopsies), Fahlén et al. obtained control samples from the terminal end of elliptical specimens taken from patients undergoing wide excision of a skin lesion [12]. Since the skin microbiome is implicated in a wide variety of dermatological conditions, it is possible that these control samples do not accurately represent a healthy microbiome. Similarly, two studies obtained unaffected samples from the region closest to the lesion [11,15], which may be affected by proximity to the lesional microbiome.

### 4.3 Microenvironment

Grice et al. surveyed 20 skin sites in 10 healthy volunteers to establish 3 distinct microenvironments associated with different microbiome profiles [18]. Owing to physiological heterogeneity in the density of hair follicles, sebaceous glands, sweat glands, moisture, exposure, pH, and temperature, among others, these cutaneous ecosystems constitute the dry (forearm, anterior knee), sebaceous (scalp, chest), and moist (intertriginous folds of the elbows and knees) microenvironments [18,19]. While sebaceous sites are dominated by *Propionibacteria* and *Staphylococci* species, moist sites are dominated by *Corynebacteria* and *Staphylococci* species, and dry sites are characterized by a mixed population of bacteria and a greater representation of Betaproteobacteria and Flavobacteriales. In addition, sebaceous sites are less diverse (including richness and evenness) than moist and dry sites [18]. The distinct microbial profiles associated with these microenvironments, as well as the fact that psoriasis commonly occurs at sites belonging to all 3 microenvironments, suggest that skin microbiome studies should include cutaneous microenvironment in their study design and/or analysis. Nevertheless, only two of the studies conducted a microenvironment-based analysis, while other studies pooled samples of the different microenvironments in the analysis [10,11]. For example, Martin et al. [15] and Gao et al. [13] included lesional samples from dry and sebaceous microenvironments which were combined in the analysis, thereby introducing a bias since the baseline characteristics of these skin sites are inherently different. Perhaps more interestingly, Alekseyenko et al. [9] and Fahlén et al. [12] attempted to account for the microenvironment in the analysis by grouping the samples according to body region, which nonetheless did not fully separate samples based on microenvironment. Alekseynko et al. noted that all samples belonged to either dry or sebaceous microenvironments, and classified samples into four categories (body, head, upper extremity, and lower extremity). Yet, a closer look at the specimen collection indicates that control elbow samples were collected from the antecubital fossa, classified as a moist microenvironment. Similarly, supplementary data shows that while samples were matched based on body region grouping (upper/lower extremity, head, and body), they were not necessarily matched according to microenvironment, such that 6 of the 51 triplets were mismatched (lesion from back [sebaceous] and control from abdomen [dry]), with 18 additional triplets if elbow samples are classified as moist. Lastly, a control site was not specified for 2 of the triplets. Perhaps microenvironment-based matching is less relevant given that the study pooled all samples in the analysis.

Fahlén ​et al. [12] employed a similar grouping system which, despite belonging to similar body regions, did not fully sort samples by the microenvironment. As such, psoriatic back (sebaceous) and flank (dry) samples, and control back and abdomen (dry) samples were grouped as “trunk”. In a similar fashion, Gao et al. [13] compared lesional samples from the dry and sebaceous microenvironments to control swabs from healthy volunteers’ forearms, representing the dry microenvironment [18].

In light of the differences between microenvironments and lack of microenvironment-based analysis in most studies, the results of these studies should be interpreted with caution. In addition, DNA collection, isolation and sequencing techniques have advanced extensively since the early studies discussed above were published, making it more challenging to compare studies over time.

Beyond the methodological considerations, a discussion of microenvironment in the study of the skin microbiome in PsD is not complete without addressing its potentially differential role in disease susceptibility. While a causal link between the skin microbiome and PsD is not established, site-specific skin microbiome has been hypothesized by some authors to play a role in triggering an immune reaction that may lead to PsA [20,21]. This hypothesis stems from the clinical observation that the *location* of psoriatic lesions is implicated in PsA susceptibility: patients with scalp or intergluteal/perianal psoriasis have a 3.89-fold or 2.35-fold increased risk to develop PsA, respectively, when compared with patients without these lesions [20]. Nevertheless, there is currently not enough evidence to conclude a causal link between the skin microbiome and PsA, nor to implicate any one microenvironment in the pathogenesis of this disease.

### 4.4 Criteria for subject selection

In addition to differences in site of collection, the studies also varied in their exclusion criteria. Only 3 of the studies excluded the use of oral antibiotics [9,11,14] and topical medications [[9], [10], [11], [12], [13], [14], [15]] (Supplementary Table 1: https://doi.org/10.17632/crhb9gdgbj.2). UV therapy, which carries an antibacterial effect, was excluded by 2 studies [12,13]. Importantly, only 5 of the studies implemented measures of psoriasis severity, most commonly the PASI score [9,11,14,15], which nevertheless ranged from 5 to 20 (Table 2). Variations in disease severity may confound the characterization of the psoriatic microbiome, in light of evidence that PASI score is significantly correlated with an isolated toxigenic strain of *S. aureus* [22].

### 4.5 Methodological considerations in interpreting genus-level results

While this review presents several trends inferred from genus-level results, it is important to highlight that these trends are preliminary. In this section, we will address two contradictions related to genus-level findings and postulate possible underlying methodological explanations.

Fahlén et al. [12] demonstrated that limb lesional samples have a lower relative abundance of *Staphylococcus* in comparison to control, a finding that contrasts the trend presented in this review (inferred from the findings of Gao et al. and Martin et al. [[13], [15]], Supplementary Table 2: https://doi.org/10.17632/crhb9gdgbj.2). This difference may be explained by several factors. First, in their analysis of limb lesions, Fahlén ​et al. obtained lesion and control samples from dry sites *only*, while both Gao et al. and Martin et al. included both dry and sebaceous lesional samples, whose physiological enrichment in *Staphylococci* may drive the observed increase in lesional relative abundance. In addition, while Martin et al. stated that control samples were site-matched (albeit with no supporting data), Gao et al. included control samples of the dry microenvironment only, increasing the likelihood that their observed increase in *Staphylococcus* relative abundance was driven by variations in microenvironment. All in all, it is plausible that the reported differences in lesional *Staphylococcus* relative abundances by these three groups were driven by the physiological enrichment in *Staphylococci* that characterizes sebaceous sites. Second, the statistical significance of the findings reported by both Gao et al. and Martin et al. is not stated. Lastly, the swabs employed by these groups retrieve surface-level bacteria, contrasting with biopsy sampling employed by Fahlén et al.; in addition, control biopsies may not represent a healthy microbiome, as described above (section 4.2).

This paper concludes that the relative abundance of Actinobacteria is lower in psoriatic compared with healthy skin, while also demonstrating a trend of an increase in *Corynebacterium* (phylum Actinobacteria) in lesions. Similarly to the discussion above, this genus-level trend is driven by the findings of Martin et al. and Gao et al., whose level of statistical significance is not stated. In addition, Martin et al. compared psoriatic lesions with atopic dermatitis samples rather than healthy control skin as in other papers, posing a challenge in comparison of findings across studies. Lastly, considering the unknown significance of these genus-level findings, it is possible that the discrepancy between the Actinobacteria and *Corynebacterium* results stems from the higher statistical power of phylum- versus genus-level taxa findings.

## 5 Conclusion

The psoriatic microbiome is characterized by a decreased alpha diversity [9,12,14,16], higher relative abundances of Firmicutes [9,[13], [14], [15], [16]] and lower relative abundances of Actinobacteria [[12], [13], [14],^16^] compared to healthy controls. Less conclusive were genus-level results, which nonetheless demonstrate trends towards increased *Streptococcus* [12,13,16], *Staphylococcus* [13,15], and *Corynebacterium* [13,15] and decreased *Propionibacterium* [12,13,16] in lesions vs. control.

## Conflicts of interest

None declared.

## Prior presentation

Canadian Rheumatology Association-Arthritis Health Professions Association Annual Scientific Meeting 2018 (poster).

## Funding sources

This work was supported by The Arthritis Society PhD Salary Award, Queen Elizabeth II: Edward Dunlop Foundation Scholarships in Science and Technology, and a donation to the Psoriatic Disease Research Program from the 10.13039/501100004089Krembil Foundation.
