# Supplementary material for: The skin microbiome in psoriatic disease: A systematic review and critical appraisal
Source: J Transl Autoimmun. 2019 Aug 20;2:100009. doi: 10.1016/j.jtauto.2019.100009 (PMC7388378; doi:10.1016/j.jtauto.2019.100009)
Supplement: Multimedia Component 1 [file mmc1.docx]

**Online-Only Supplements**

This document contains the following appendices:

- **Appendix 1: Search Strategy**
  - **eTable 1**: Database(s): Ovid MEDLINE(R) Epub Ahead of Print, In-Process & Other Non-Indexed Citations, Ovid MEDLINE(R) Daily and Ovid MEDLINE(R), 1946 to Present Search Strategy
  - **eTable 2**: Database(s): Embase 1974 to 2018 January 23 Search Strategy:
  - **eTable 3**: Database(s): EBM Reviews - Cochrane Central Register of Controlled Trials December 2017 Search Strategy:
- **eMethods:** Inclusion and Exclusion Criteria
- **eTable 4:** Risk of Bias Analysis Tool (Adapted from Hamidi *et al*.^1^)

**Appendix 1: Search Strategy**

The databases searched were:

**eTable 1: Database(s): Ovid MEDLINE(R) Epub Ahead of Print, In-Process & Other Non-Indexed Citations, Ovid MEDLINE(R) Daily and Ovid MEDLINE(R), 1946 to Present Search Strategy:**

| **#** | **Searches** |
| --- | --- |
| 1 | exp Psoriasis/ |
| 2 | pustulos*.mp. |
| 3 | psoria*.mp. |
| 4 | Acrodermatitis continua.mp. |
| 5 | parapsoria*.mp. |
| 6 | 1 or 2 or 3 or 4 or 5 |
| 7 | exp Microbiota/ |
| 8 | exp Bacteria/ |
| 9 | flora.mp. |
| 10 | microflora*.mp. |
| 11 | bacteri*.mp. |
| 12 | microb*.mp. |
| 13 | micro-bio*.mp. |
| 14 | microorganism*.mp. |
| 15 | micro-organism*.mp. |
| 16 | metagenom*.mp. |
| 17 | meta-genom*.mp. |
| 18 | 7 or 8 or 9 or 10 or 11 or 12 or 13 or 14 or 15 or 16 or 17 |
| 19 | 6 and 18 |
| 20 | cutane*.mp. |
| 21 | epicutane*.mp. |
| 22 | supracutane*.mp. |
| 23 | cutis*.mp. |
| 24 | exp Skin/ |
| 25 | skin.mp. |
| 26 | derm*.mp. |
| 27 | epiderm*.mp. |
| 28 | integument*.mp. |
| 29 | tegument*.mp. |
| 30 | 20 or 21 or 22 or 23 or 24 or 25 or 26 or 27 or 28 or 29 |
| 31 | 19 and 30 |
| 32 | limit 31 to english language |
| 33 | exp animals/ not (exp animals/ and exp humans/) |
| 34 | 32 not 33 |

**eTable 2: Database(s): Embase 1974 to 2018 January 23 Search Strategy:**

| **#** | **Searches** |
| --- | --- |
| 1 | exp psoriasis/ |
| 2 | psoriatic arthritis/ |
| 3 | pustulos*.mp. |
| 4 | psoria*.mp. |
| 5 | Acrodermatitis continua.mp. |
| 6 | parapsoria*.mp. |
| 7 | 1 or 2 or 3 or 4 or 5 or 6 |
| 8 | exp microflora/ |
| 9 | exp bacterium/ |
| 10 | flora.mp. |
| 11 | microflora.mp. |
| 12 | bacteri*.mp. |
| 13 | microb*.mp. |
| 14 | micro-bio*.mp. |
| 15 | microorganism*.mp. |
| 16 | micro-organism*.mp. |
| 17 | metagenom*.mp. |
| 18 | meta-genom*.mp. |
| 19 | 8 or 9 or 10 or 11 or 12 or 13 or 14 or 15 or 16 or 17 or 18 |
| 20 | 7 and 19 |
| 21 | exp skin/ |
| 22 | cutane*.mp. |
| 23 | epicutane*.mp. |
| 24 | supracutane*.mp. |
| 25 | cutis*.mp. |
| 26 | skin.mp. |
| 27 | derm*.mp. |
| 28 | epiderm*.mp. |
| 29 | integument*.mp. |
| 30 | tegument*.mp. |
| 31 | 21 or 22 or 23 or 24 or 25 or 26 or 27 or 28 or 29 or 30 |
| 32 | 20 and 31 |
| 33 | limit 32 to english language |
| 34 | (exp animals/ or exp animal experimentation/ or nonhuman/) not ((exp animals/ or exp animal experimentation/ or nonhuman/) and exp human/) |
| 35 | 33 not 34 |
| 36 | remove duplicates from 35 |

**eTable 3: Database(s): EBM Reviews - Cochrane Central Register of Controlled Trials December 2017 Search Strategy:**

| **#** | **Searches** |
| --- | --- |
| 1 | exp Psoriasis/ |
| 2 | pustulos*.mp. |
| 3 | psoria*.mp. |
| 4 | Acrodermatitis continua.mp. |
| 5 | parapsoria*.mp. |
| 6 | 1 or 2 or 3 or 4 or 5 |
| 7 | exp Microbiota/ |
| 8 | exp Bacteria/ |
| 9 | flora.mp. |
| 10 | microflora*.mp. |
| 11 | bacteri*.mp. |
| 12 | microb*.mp. |
| 13 | micro-bio*.mp. |
| 14 | microorganism*.mp. |
| 15 | micro-organism*.mp. |
| 16 | metagenom*.mp. |
| 17 | meta-genom*.mp. |
| 18 | 7 or 8 or 9 or 10 or 11 or 12 or 13 or 14 or 15 or 16 or 17 |
| 19 | 6 and 18 |
| 20 | cutane*.mp. |
| 21 | epicutane*.mp. |
| 22 | supracutane*.mp. |
| 23 | cutis*.mp. |
| 24 | exp Skin/ |
| 25 | skin.mp. |
| 26 | derm*.mp. |
| 27 | epiderm*.mp. |
| 28 | integument*.mp. |
| 29 | tegument*.mp. |
| 30 | 20 or 21 or 22 or 23 or 24 or 25 or 26 or 27 or 28 or 29 |
| 31 | 19 and 30 |

**eMethods: Inclusion and Exclusion Criteria**

*Inclusion criteria:*

Studies on psoriasis and/or psoriatic arthritis that investigated the microbiome of the psoriatic plaque using culture-independent sequencing technology

Next generation sequencing, target sequencing of 16S rRNA gene

*Exclusion criteria:*

Other dermatological diseases

Psoriasis studies employing culture-based techniques

Metagenomic studies

Mass spectrometry studies

**eTable 4: Risk of Bias Analysis Tool (Adapted from Hamidi *et al*.^1^)**

| **Bias domain** | **No Risk of Bias if** |
| --- | --- |
| Sample selection | Consecutive unselected population of PsD patients  PsD primary study group  Consecutive PsD patients as study population  PsA classification criteria applied  Criteria for cases and controls explained |
| Exposure Assessment | Chronic plaque psoriasis diagnosed by a dermatologist  PsA disease related and associated features assessed |
|  |  |
| Confounding Factors | Cases and controls compared after adjusting for age and sex  Unaffected sample obtained from contralateral site  Microenvironment was matched between psoriasis and control samples  Antibiotics, topical medication, and UV therapy excluded |
| Selective Outcome Reporting | Alpha and beta diversity reported  Relative taxa abundance reported for study population and controls, including significance of differences between the groups, if any |
| Attrition Bias | Percentage of attrition present  All subjects from the start to the end accounted for in the results |
| Analytical assessment | Appropriate statistical analysis conducted  Sample size calculation performed, and adequate sample included for the study |

Risk of Bias was:

Low- if No risk of bias in at least 5 of the seven domains

Moderate- if No risk of bias in three or four domains

High- if No risk of bias in two or less domains

**References**

1. Hamidi M, Boucher BA, Cheung AM, et al. Fruit and vegetable intake and bone health in women aged 45 years and over: a systematic review. Osteoporos Int. 2011;22(6):1681-93.
